# Investigations into Capillary Forces and Capillary Rise in a Three-Finger Microgripper and a Plate: Numerical Simulations and Experimental Validation

**DOI:** 10.3390/mi15121459

**Published:** 2024-11-29

**Authors:** Zhi Xu, Zenghua Fan, Jun Gao, Kun Zhang, Xiang Zhang

**Affiliations:** School of Mechanical Engineering, Shandong University of Technology, Zibo 255049, China

**Keywords:** capillary bridge, energy minimization, capillary forces, capillary rise, three-finger microgripper

## Abstract

The assembly and position adjustment of micro-components have wide applications in micro-electromechanical systems, wafer packaging and biomedicine. However, current single-finger microgrippers only allow for the pickup and release of micro-components. In the present study, a three-finger capillary microgripper was developed to pick up, release and adjust the position of micro-components. The capillary force and the capillary rise generated by the capillary bridge were investigated by simulation and experiments. A simulation model was set up by the minimum energy method. On the established experimental platform, capillary forces were measured at different separation distances. When the volume was 0.9 μL, the maximum capillary forces gained from the capillary bridge model and experiments were 95.2 μN and 96.0 μN, respectively. A comparison of the capillary bridge models and the experimental results of the capillary forces demonstrate the reliability of the capillary bridge models. The influences of various parameters were investigated in detail by the capillary bridge model. The results demonstrate that when the side of the probe is hydrophilic, the variations in the capillary force with various factors such as separation distance and capillary bridge volume is non-monotonic, which is caused by the restriction of the probe edge.

## 1. Introduction

With the development of miniaturization and the increased precision of products in biomedical and microelectronic fields, the flexible pick-and-place of micro-components is a fundamental task. Various traditional microgrippers [1,2,3,4] have limited applications due to their own shortcomings. For example, clamp-on microgrippers [5] cannot be applied to soft micro-components due to the presence of mechanical stresses. Microgrippers based on vacuum adsorption [6] are barely suitable for handling irregular objects. Several non-traditional micromanipulation methods have been investigated, such as optical tweezers [7,8], electrokinetic tweezers [9,10,11] and acoustic tweezers [12,13]. The capillary microgripper enables the smooth handling and orientation adjustment of micro-components and have attracted much attention in microscale manipulation.

A variety of capillary microgrippers have been developed to achieve the pickup and release of micro-components. Fan et al. [14,15] reported a microgripper made using a hydrophobic single probe, in which the pickup and release of micro-components were achieved by a dropwise condensation approach. Vasudev et al. [16] successfully designed an electrowetting microgripper for picking up and releasing glass beads with a gravity of 77–136 μN. Hagiwara et al. [17] designed a microgripper with a piston to control droplets. The micro-parts of cones, cubes and half-cylinders at a sub-millimeter scale were picked up and placed reliably. By studying the effect of the different shapes of probes on the capillary force, Saito et al. [18] found that a concave probe provided a large capillary force. Iazzolino et al. [19] investigated the effect of liquid bridge volume on capillary force and proposed a microgripper that controls capillary force by controlling the liquid bridge volume. Fantoni et al. [20] designed a microgripper to control the capillary force by changing the contact angle between the capillary bridge and the micro-component. A flat surface was changed to a hemispherical shape based on inflatable membrane management [21]. The capillary forces were controlled by changing the surface to achieve the micro-component grip. Takatoshi et al. [22] developed a single-nozzle capillary microgripper integrated with a visual feedback system to adjust the micro-components in real time. Cavaiani et al. [23] designed a capillary gripper made by combining stereolithography. The capillary gripper was used to pick up and release sub-millimetric surface-mounted devices (SMD).

Multi-finger capillary microgrippers have been developed to control the position of micro-components during manipulation processes. The relationship between the position of the micro-components and the relative position of each probe was established to control the micro-components [24]. Tanaka et al. [25] described a capillary microgripper with two nozzles for the manipulation of cubical triangular prismatic and helical 1 mm-sized objects. Fan et al. [26] investigated the capillary forces between hydrophobic probes and a plate by the energy minimization method. Comparison investigations were conducted between single-finger and a three-finger microgripper, which demonstrated that a three-probe capillary bridge can provide a greater capillary force than a single-probe capillary microgripper. Investigations into capillary forces are critical for capillary microgrippers. Sun et al. [27] studied liquid bridges among spherical particles by using the volume-of-fluid, immersed-boundary, direct-numerical-simulation (VOF-IB-DNS) method. A theoretical equation was proposed for calculating the capillary forces between spherical particles and rough planes [28]. Iwamatsu et al. [29] researched the effect of line tension on the vapor–liquid equilibrium of capillary bridges using capillary theory. Semprebon et al. [30] investigated liquid morphologies and capillary forces between three spherical beads by surface energy minimization using Surface Evolver. The capillary bridge model between the non-flush three-finger microgripper and a plate was established by Fan et al. [31] through the method of energy minimization. The effect of parameters such as separation distance and radial distance on the capillary force was analyzed in detail by the model. Yang et al. [32] discussed the effect of particle size ratio, contact angle and separation distance on the capillary force using a mathematical model, and the results show that both the capillary force and van der Waals force increase with particle size. The capillary bridges between two circular disks were investigated by simulation and experiments [33]. The influence of the radius ratio of the two circular disks on the capillary force was analyzed. Chau et al. [34] proposed a three-dimensional model for calculating the capillary force in capillary condensation. The importance of tilt angles was investigated. Wang et al. [35] investigated the contact angle of a droplet on homogeneous and rough substrates by developing the generalized Young’s equation.

Although single-finger and multi-finger capillary microgrippers have been developed for the manipulation of micro-components, the capillary force of multi-finger capillary microgrippers caused by capillary rise has not been considered. In the present study, a three-finger microgripper with a hydrophilic side was researched. A capillary bridge was obtained in the three probes and an acrylic plate. The capillary force and the capillary rise were recorded using the experimental platform. The capillary force model was established between the three probes and the plate. The experimental results verify the validity of the capillary bridge model and the capillary force theory solution method. The capillary bridge on the probes with the hydrophilic surface may provide a reference for developing a new method to control micro-components.

## 2. Capillary Bridge Model

**Figure 1:**
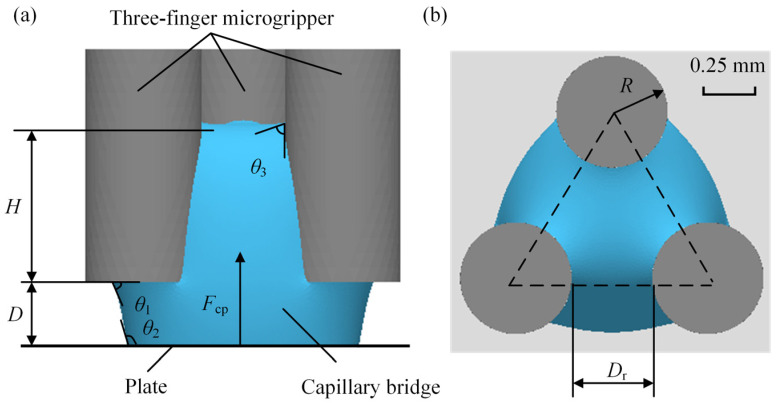
Capillary bridge between the microgripper and the plate: (**a**) front view, (**b**) top view.

The capillary bridge on the end face of the three probes is shown in Figure 1. *D* is the separation distance, and *θ*_1_ is the contact angle on the end face of the microgripper, as shown in Figure 1a. *θ*_2_ is the plate contact angle, and *θ*_3_ is the probe side contact angle. The apparent contact angles were considered in the model. *D*_r_ is the radial distance between the probes, *R* is the radius of the probes and *H* is the capillary rise height. The microgripper is composed of three cylindrical probes with an equilateral triangle shape, as shown in Figure 1b. The capillary force consists of the surface tension and the differential hydrostatic pressure. The capillary force was obtained by the equation [36]

(1) $$F_{\mathrm{cp}}=\gamma_{\mathrm{lg}}l\sin\theta_{2}-\Delta pA_{1}$$

where *γ*_lg_ and *θ*_2_ are the liquid–gas interfacial tension and the plate contact angle, respectively; Δ*p* is the pressure difference, *l* is the three-phase contact line and *A*_1_ is the contact area on the plate.

## 3. Experiments and Simulations

### 3.1. Experimental Setup

**Figure 2:**
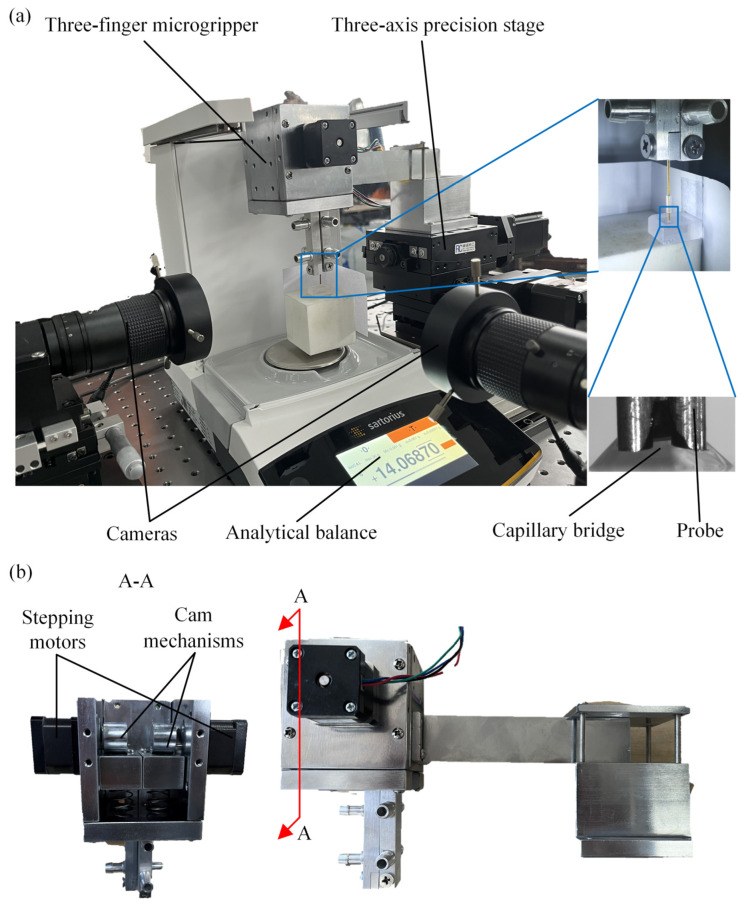
Experimental setup: (**a**) measurement platform, (**b**) structure of the three-finger microgripper.

An experiment setup was constructed to investigate the capillary bridges, as depicted in Figure 2. The analytical balance was used to measure the capillary force. The measurement accuracy and the maximum range of the analytical balance were 0.1 μN and 0~0.15 N, respectively. Two high-resolution industrial cameras (Myutron HMZ0745, Tokyo, Japan) and a three-axis precision stage were used to record the capillary bridge and move the three probes, respectively, as shown in Figure 2a. The movements of the three-finger microgripper were achieved by the three-axis precision stage. The structure of the three-finger microgripper is shown in Figure 2b. The three-finger microgripper is composed of stepping motors, cam mechanisms and probes. The movements of the cam mechanisms are achieved by the stepping motors. The probes are fixed at the end of the cam mechanisms. The relative motions between the three probes are realized by the cam mechanisms. The capillary force measurements were performed on an optical table to minimize measurement errors. Three probes were secured to the end of the three-finger microgripper. A capillary bridge was established between the three probes and the plate. Moreover, the experiment setup was placed in a clean room to prevent the liquid from being contaminated at a temperature of 24 ± 1 °C.

### 3.2. Experimental Methods

To reduce errors resulting from liquid evaporation, a 50% aqueous glycerin solution was adopted in the experiments. The surface tension of the mixture liquid was 67.4 mN/m. The probes were made of copper wires with a diameter of 0.5 mm. An acrylic plate was used as the substrate. A droplet was placed on the plate by a pipette (TopPette, Dragonlab, Beijing, China). The apparent contact angle with a droplet volume of 0.9 μL was measured by a contact angle goniometer (JC2000D1, POWEREACH, Shanghai, China). The apparent contact angles on the copper surface and the acrylic plate were 62° and 56°, respectively. ImageJ open software (ImageJ 1.54d) was applied to measure the liquid–solid contact angles based on the obtained experimental images. The apparent contact angles from the pull-off measurement were obtained from the experimental images. The capillary force was predicted based on the obtained apparent contact angles in the established model. The motion of the microgripper was achieved by the three-axis precision stage. The contact and the capillary bridge were established by moving the probe downward. The capillary rise phenomenon was observed in the probes because the sides of the probes were hydrophilic. The three-finger microgripper stretched the capillary bridges upward under the control of the three-axis precision stage. The speed of the three-finger microgripper was 10 μm/s, and the distance of each upward stretch was 20 μm. The variation in the capillary bridge and the capillary rise height was caused by the stretching of the capillary bridge. After each stretch was completed, the number of indications was recorded when the analytical balance was stable. The capillary force *F*_cp_ in a steady state was obtained by

(2) $$F_{\mathrm{cp}}=(m_{0}-m_{n})g$$

where *m*_0_ is the initial indication of the analytical balance, *m*_n_ is the reading of the analytical balance and *g* is the acceleration of gravity. In the experiments, the measuring procedure was repeated three times to reduce the measurement errors.

### 3.3. Comparison Between the Simulation and the Experiment

**Figure 3:**
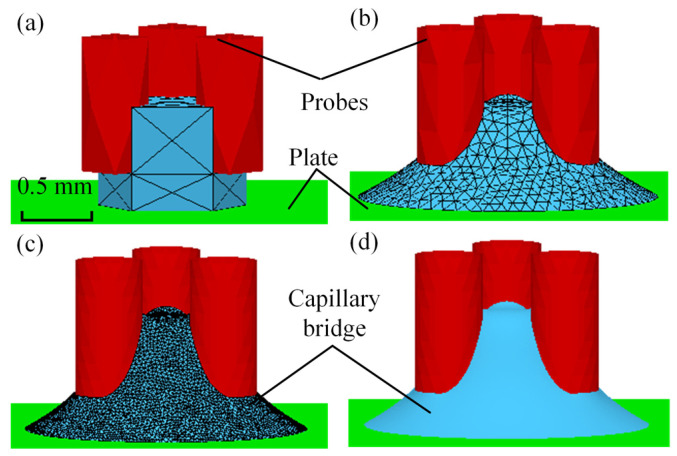
Capillary bridge evolution process: (**a**) initial skeleton, (**b**) refinement evolution, (**c**) evolution capillary bridge, (**d**) capillary bridge shape after evolution.

The numerical model was constructed in Surface Evolver (SE) to simulate the wetting state of the capillary bridge based on energy minimization. Various stabilization capillary bridge systems have been investigated using the energy minimization method [37,38,39]. The model was constructed by setting parameters, building the initial skeleton, defining the boundary conditions and performing the surface evolution. The radius of the probes, the radial distance between the probes, the contact angle and the volume of the capillary bridge are defined as parameters in the capillary bridge model. Shape constraints and energy minimization constraints are defined as boundary conditions. The shape of the plate and the probes were constrained by the equations of the plane and the equations of the circle. The initial skeleton was formed of vertices, edges and facets. After the definition, the capillary model performed the surface evolution by the energy minimization method with a constant volume. The capillary bridge length was defined as $l_{c}=\sqrt{\gamma /\rho g}$, where *ρ* is the density and *g* is the acceleration of gravity. In the experiments, *l*_c_ = 2.53 mm and the capillary bridge radii are 0.56 mm. The capillary bridge length is larger than the capillary bridge radii. The capillary effect becomes a dominant factor compared with the gravity effect at microscale. Therefore, gravity is not considered in capillary bridge models. The total energy *E* of the capillary bridge system is [33,40,41]

(3) $$E=A_{sl}\gamma_{sl}+A_{sg}\gamma_{sg}+A_{lg}\gamma_{lg}$$

where *A*_sl_, *A*_sg_ and *A*_lg_ are the areas of the solid–liquid, solid–gas and liquid–gas interfaces, respectively, and *γ*_sl_, *γ*_sg_ and *γ*_lg_ are the surface tension of the solid–liquid, solid–gas and liquid–gas interfaces, respectively. The total energy is the sum of the three interfacial energies. The calculation processes of the capillary bridge model are depicted in Figure 3. The initial shape of the geometry is established by defining the geometrical elements, as shown in Figure 3a. The model was refined in the mesh according to the relevant parameters being defined, as shown in Figure 3b. The stable capillary bridge was obtained when the absolute difference in the total energy *E* between two iterations was less than 10^−6^, as shown in Figure 3c. The shape of the evolved complete capillary bridge is shown in Figure 3d. The capillary forces were calculated based on the obtained contact angle *θ*_2_, the pressure difference ∆*p*, the contact line on plate *l* and the contact area on plate *A*_1_, according to Equation (1).

**Figure 4:**
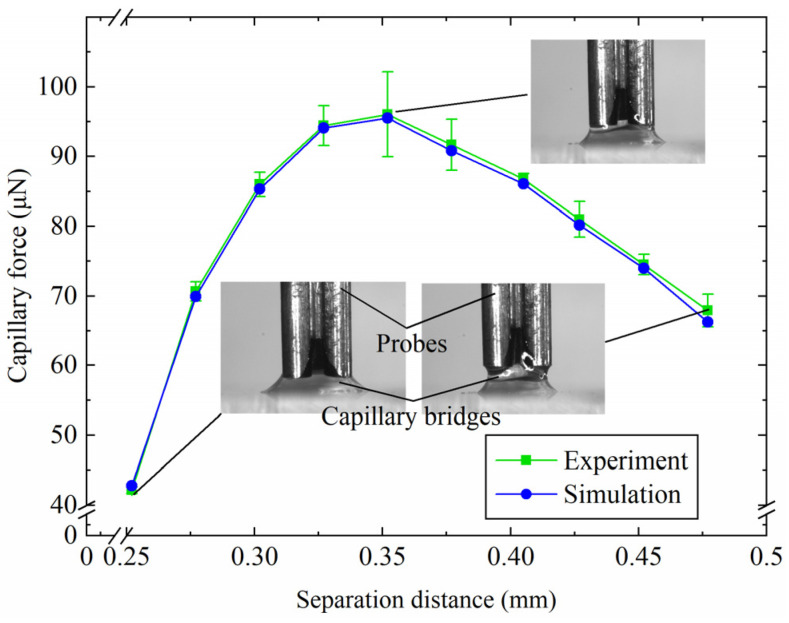
Capillary forces in the experiment and the capillary bridge model.

**Figure 5:**
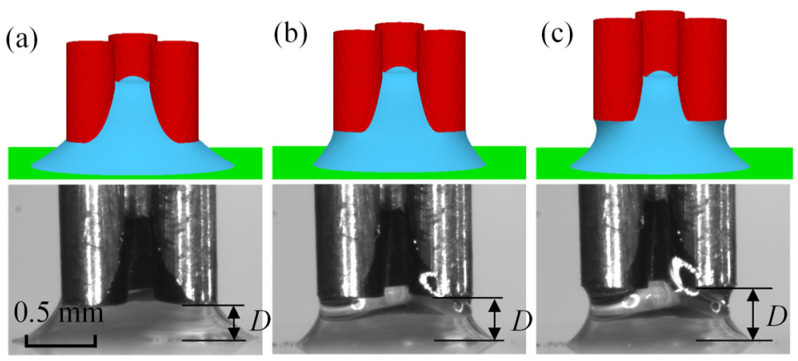
Capillary bridges in the capillary bridge model and the experiments: (**a**) *D* = 0.28 mm, (**b**) *D* = 0.38 mm, (**c**) *D* = 0.45 mm.

Figure 4 shows the capillary forces with various separation distances *D* for the capillary bridge model and the experimental results. The capillary force increases and then decreases with the increase in the separation distance. For *V* = 0.9 μL, the maximum capillary forces of the capillary bridge model and the experiment were 95.2 μN and 96.0 μN, respectively. An increase in the separation distance led to an increase in the capillary bridge curvature, which caused the increase in the capillary force. The capillary force reaches a maximum value and then starts to decrease, which was caused by the disappearance of the edge effect. The simulation model was established based on the assumption that there was no angle hysteresis, which resulted in a smaller capillary force in the simulation than in the experiments. The measured contact angles were defined in the capillary bridge model parameter settings to reproduce the capillary bridge in the experiments. Figure 5 shows the capillary bridge shape in the model and the experiments with different separation distances. The geometry of the capillary bridge was predicted successfully by the established model for a separation of 0.28 mm, as shown in Figure 5a. Figure 5b,c shows the experimental and model-predicted capillary bridge shapes with separation distances of 0.38 mm and 0.45 mm, respectively.

## 4. Results and Discussion

In this paper, the influence of various factors on the capillary force and the capillary height were researched based on a model. The apparent contact angles were defined in the capillary bridge model. In addition, the acrylic plate was assumed to be a smooth and homogenous surface.

### 4.1. Influence of Separation Distances

**Figure 6:**
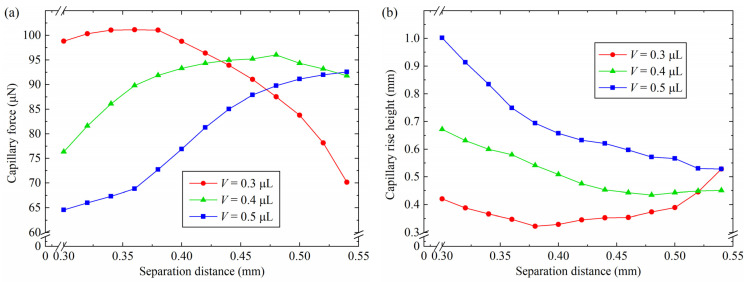
Influence of the separation distance on the capillary force and the capillary rise height: (**a**) capillary force, (**b**) capillary rise height.

Simulation models were established to research the influence of the separation distance on the capillary force and the capillary rise height. The parameters were defined as shown in Table 1. Figure 6 shows the changes in the capillary force and the capillary rise height by varying the separation distance. For *V* = 0.3 μL and 0.3 mm ≤ *D* ≤ 0.36 mm, the capillary force increased with the increase in separation distance, as shown in Figure 6a. For 0.36 mm < *D* ≤ 0.54 mm, the capillary force decreased with the increasing separation distance. For *V* = 0.4 μL, the capillary force increased from 76.4 μN to 96.0 μN and then decreased to 91.8 μN. The maximum capillary force was obtained with a separation distance of 0.48 mm. The trends in capillary force change were similar when the capillary bridge volume was 0.3 μL and 0.4 μL. For *V* = 0.5 μL and 0.3 mm < *D* ≤ 0.54 mm, the capillary force increased as the separation distance increased, which shows a different trend from a capillary bridge volume of 0.3 μL and 0.4 μL. When the capillary bridge volume was constant, the increase in separation distance contributed to a decrease in the capillary bridge curvature and an increase in the radius of curvature. The capillary bridge changed from a convex shape to a concave one. According to the Young–Laplace equation, the surface curvature of the capillary bridge is proportional to the pressure difference of the capillary bridge. The reduction in pressure difference led to a large capillary force, in accordance with Equation (1). As the separation distance increased further, the three probes’ end face could not be infiltrated by the capillary bridge, which caused the disappearance of the edge effects. The edge of the probes was unable to restrict the capillary bridge motion on the three probes’ end face. The three-phase contact line decreased with the increasing separation distance. According to Equation (1), a reduction in capillary force was generated. The variation in the capillary rise height with different separation distances is shown in Figure 6b. The capillary rise height reduced with the increasing separation distance for *V* = 0.5 μL. For *V* = 0.3 μL, the capillary rise height reduced to 0.3 mm from 0.4 mm and then increased to 0.5 mm.

### 4.2. Influence of the Capillary Bridge Volume

**Figure 7:**
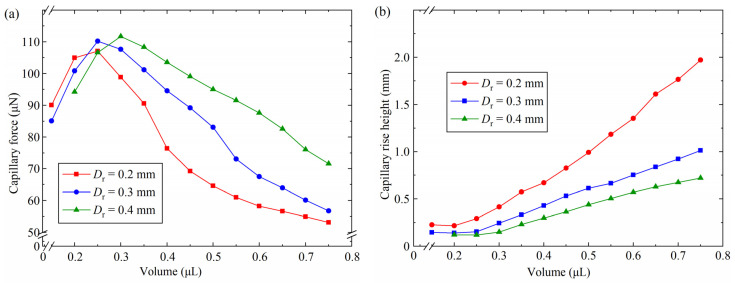
Effect of the capillary bridge volume on the capillary force and the capillary rise height: (**a**) capillary force, (**b**) capillary rise height.

To investigate the influence of the capillary bridge volume on the capillary bridge force and the capillary rise height, simulations were performed with the constant shown in Table 2. The variation in the capillary force and the capillary rise height with the capillary bridge volume is shown in Figure 7. For *V* = 0.15 μL and *D*_r_ = 0.4 mm, a rupture of the capillary bridge was observed. For 0.15 μL < *V* < 0.25 μL, the capillary force increased with the increase in the capillary bridge volume, as shown in Figure 7a. For *V* < 0.25 μL, the capillary bridge was unable to fully infiltrate the three-finger microgripper end face, which caused the absence of edge effects. Consequently, the contact area on the end face of the three probes and length of the three-phase contact line increased as the volume increased. In the absence of edge effects, as the volume increased, the width of the capillary bridge also increased. The increase in the volume and the width of the capillary bridge resulted in an increase in the length of the three-phase contact line of the capillary bridge, which caused an increase in the capillary force. For *D*_r_ = 0.2 mm, 0.3 mm and 0.4 mm, the maximum capillary forces were 107.0 μN, 110.2 μN and 111.7 μN, respectively. For 0.3 μL < *V* < 0.75 μL, the capillary force reduced with the increase in the capillary bridge volume. The minimum capillary forces were 53.1 μN, 56.7 μN and 71.5 μN with *D*_r_ = 0.2 mm, 0.3 mm and 0.4 mm, respectively. The three-phase contact line was restricted on the end face of the three-finger microgripper, as the capillary bridge volume increased further. The convex shape of the capillary bridge was obtained, which resulted in an increase in the pressure difference. The reduction in the capillary force was caused by the increase in the pressure difference. The capillary bridge became a convex capillary bridge, resulting in an increase in the pressure difference and thus a decrease in the capillary force, when the capillary bridge volume was 0.4 μL. In addition, for *V* = 0.5 μL, the capillary forces were 64.6 μN, 83.1 μN and 94.9 μN with radial distances of 0.2 mm, 0.3 mm and 0.4 mm, respectively. The largest capillary force was gained with radial distances of 0.4 mm. A small capillary rise height was obtained for *D*_r_ = 0.4 mm, as shown in Figure 7b. For *D*_r_ = 0.2 mm, 0.3 mm and 0.4 mm, the minimum capillary rise height resulted in the largest capillary bridge volume between the end face of the three probes and the plate. Consequently, the capillary bridge had a large curvature radius, a small curvature and a long three-phase contact line, which led to a large capillary force.

### 4.3. Influence of the Radial Distance Between Probes

**Figure 8:**
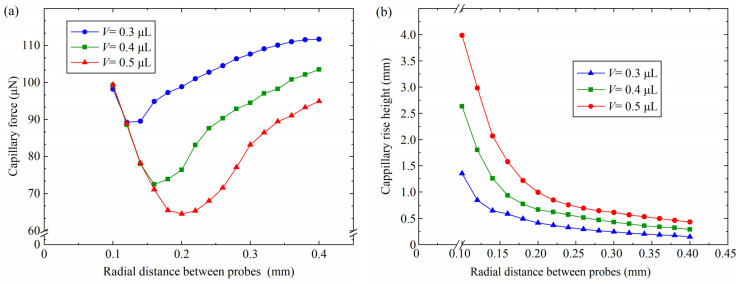
Effect of the radial distance between probes on the capillary force and the capillary rise height: (**a**) capillary force, (**b**) capillary rise height.

The simulation parameters in Table 3 were used to investigate capillary bridges with different radial distances. The relationships between the capillary force, the capillary rise height and the radial distance between probes were investigated, as shown in Figure 8. For 0.1 mm < *D*_r_ < 0.14 mm, the capillary force decreased with the increase in the radial distance between probes, as shown in Figure 8a. The capillary force reduced to 72.4 μN from 99.2 μN as the radial distance between the probes increased from 0.1 mm to 0.16 mm. The capillary bridges completely infiltrated the three-finger microgripper end face for *D*_r_ < 0.12 mm and *V* = 0.5 μL. The capillary bridge curvature improved with the increase in the radial distance between the probes in the case of edge effect. The pressure difference in the capillary bridges increased with the increasing capillary bridge curvature. According to Equation (1), the increase in the capillary bridge pressure difference caused a decrease in the capillary force. However, the capillary force increased as the radial distance of the probes increased from 0.2 mm to 0.4 mm. For *V* = 0.5 μL, the capillary force increased to 95.0 μN from 64.6 μN as the radial distance increased further. The edge effect disappeared when *V* = 0.5 μL and *D*_r_ = 0.2 mm. The end face of the probes was not infiltrated by the capillary bridges, which led to a decrease in the curvature. The contact area on the three probes’ end face decreased with the increasing radial distance between the probes, which caused the capillary bridge curvature to decrease. The decrease in the pressure difference led to an increase in the capillary force. In addition, for *D*_r_ = 0.26 mm, the capillary forces were 104.5 μN, 90.3 μN and 71.5 μN with a volume of 0.3 μL, 0.4 μL and 0.5 μL, respectively. The largest capillary force was gained by a volume of 0.5 μL. The capillary rise height reduced with the increase in the radial distance between probes, as shown in Figure 8b. The capillary rise height reduced to 0.43 mm from 2.98 mm with a capillary volume of 0.5 μL when the radial distance between the probes increased from 0.12 mm to 0.4 mm, The minimum capillary rise height were 0.14 mm and 0.29 mm with volumes of 0.3 μL and 0.4 μL, respectively. The liquid–gas interface and the surface tension of the liquid–gas interface increased with the increase in the radial distance between the probes, which led to a reduction in the capillary rise height.

### 4.4. Influence of the Plate Contact Angle

**Figure 9:**
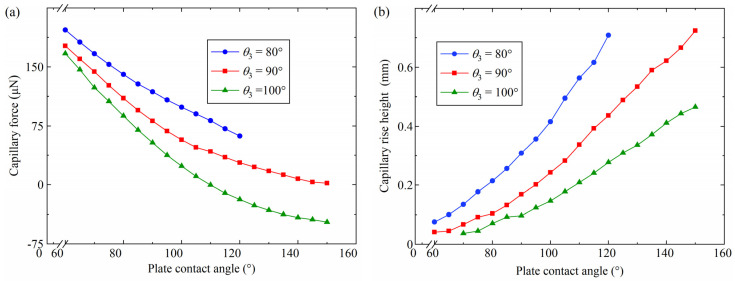
Effect of the plate contact angle on the capillary force and the capillary rise height: (**a**) capillary force, (**b**) capillary rise height.

The capillary bridges at various plate contact angles were researched using the simulation constant, as shown in Table 4. The variation in the capillary force and the capillary rise height with the plate contact angle is shown in Figure 9. Figure 9a illustrates that the capillary force reduces with the increasing plate contact angle *θ*_2_. For *θ*_3_ = 100°, the capillary force reduced to −47.2 μN from the initial 166.9 μN when the plate contact angle increased to 150° from the initial 60°. As the plate contact angle increased from 60° to 120°, the capillary force decreased from 197.0 μN to 62.0 μN for a probe side contact angle *θ*_3_ of 80°. A rupture of the capillary bridge was observed when the plate contact angle was 120° and the probe side contact angle was 80°. The capillary bridge changed to a concave shape when the plate contact angle *θ*_2_ was an acute angle. As the plate contact angle *θ*_2_ increased, the capillary bridge changed from a concave capillary bridge to a convex capillary bridge. The change in shape resulted in a large capillary bridge curvature and a great pressure difference, which led to a reduction in the capillary force. In addition, when the plate contact angle was 85°, the capillary force was 128.2 μN, 95.0 μN and 69.8 μN with a *θ*_3_ of 80°, 90° and 100°, respectively. The largest capillary force was gained with a probe side contact angle of 80°. The capillary rise height increased with an increasing plate contact angle, as shown in Figure 9b. In addition, a capillary bridge without a capillary rise was observed when the probe side contact angle *θ*_3_ was 100° and the plant contact angle was 60° and 65°. The plate changed from hydrophilic to hydrophobic as the plate contact angle increased. The capillary bridge tended to move as the plate contact angle increased, which caused an increase in the capillary rise height.

### 4.5. Influence of the Probe Side Contact Angle

**Figure 10:**
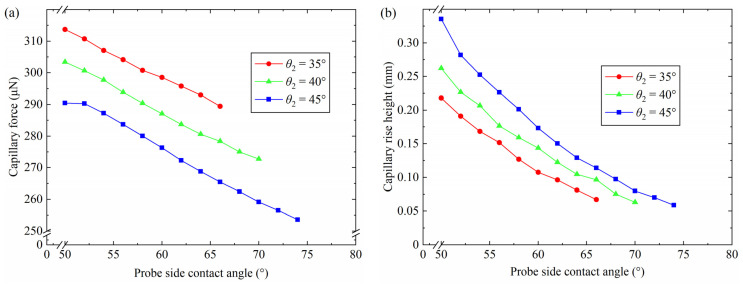
Effect of the probe side contact angles on the capillary force and the capillary rise height: (**a**) capillary force, (**b**) capillary rise height.

Capillary bridges with a different probe side contact angle *θ*_3_ were analyzed using the simulation parameters in Table 5. The variation between the capillary force, the capillary rise height and the probe side contact angle *θ*_3_ with different plate contact angles is illustrated in Figure 10. As the probe side contact angle increased from 50° to 74°, the capillary force decreased from 290.6 μN to 253.6 μN for *θ*_2_ = 45°, as shown in Figure 10a. Similarly, the capillary force reduced to 272.7 μN from 303.4 μN with a plate contact angle of 40° when the probe side contact angle increased from 50° to 70°. The maximum capillary force was 313.7 μN with a probe side contact angle of 50° and a plate contact angle of 35°. The capillary forces were 298.6 μN, 283.7 μN and 276.3 μN with plate contact angles of 35°, 40° and 45°, respectively, for *θ*_3_ = 60°. The largest capillary force was gained with a plate contact angle of 35°. In addition, a rupture of the capillary bridge was observed when the plate contact angle was 35° and the probe side contact angle was 66°. The results show that the capillary bridge breaks were caused by a small plate contact angle. The variations in the capillary rise height have an influence on the morphology of the capillary bridge, causing changes in the capillary force. Figure 10b shows that the capillary rise height decreased with an increasing probe side contact angle. The hydrophilicity of the probe side surface reduced with the increase in the probe side contact angle, which caused a reduction in the capillary rise height. The decrease in the capillary rise height led to an increase in the capillary bridge volume between the end face of the three probes and the plate. The increase in the capillary bridge volume between the end face of the three probes and the plate changed the capillary bridge into a convex one, which increased the pressure difference. In addition, the contact area on the acrylic plate added to the reduction in the capillary rise height. According to Equation (1), the reduction in the capillary force was due to the increase in the pressure difference and the contact area on the acrylic plate.

### 4.6. Pickup and Release Experiments

**Figure 11:**
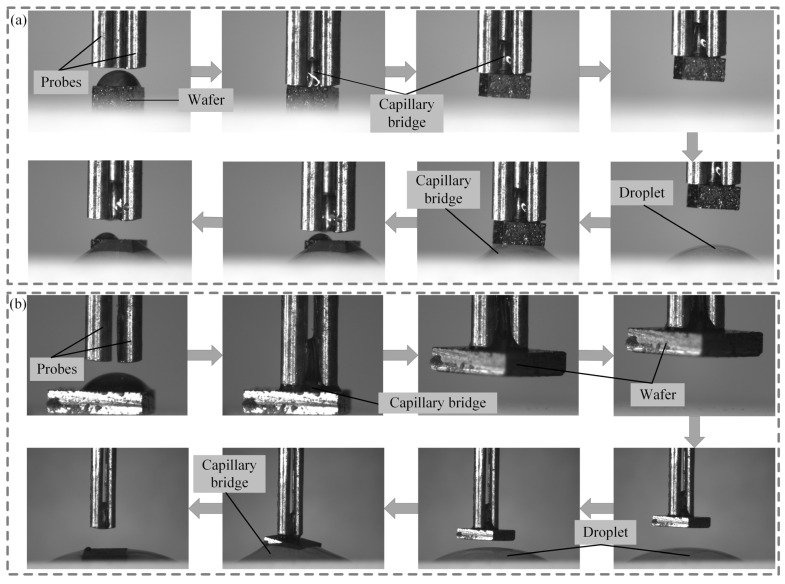
Pickup and release of micro-components: (**a**) 1 mm × 1 mm × 0.8 mm, (**b**) 2 mm × 2 mm × 0.625 mm.

The micro-component pickup and release experiments were performed based on the established experimental platform. Two different sizes of wafers were picked up and released using the three-finger microgripper, as shown in Figure 11. Wafers of 1 mm × 1 mm × 0.8 mm^3^ and 2 mm × 2 mm× 0.625 mm were placed on the acrylic plate. The three-finger microgripper was moved over the wafer by controlling the three-axis precision stage, as shown in Figure 11a. The volume of the droplet on the wafer was 0.5 μL. The capillary bridge between the probes and the wafer was formed by moving the three-finger microgripper downward, and capillary rise was observed due to the radial distance between the probes. As the three-finger microgripper moved upward at a given speed of 20 μm/s, the wafer with a size of 1 mm × 1 mm × 0.8 mm was picked up successfully. Then, the wafer was transferred to the target position by controlling the three-axis precision stage. Aqua distillate with a volume of 0.7 μL was placed in the target position. The three-finger microgripper moved downward to release the wafer. A new capillary bridge between the three-finger microgripper and the plate was formed. Finally, the wafer was released in the target position successfully. To further investigate the pickup and release ability of the three-finger microgripper, a micro-component test was also performed on a 2 mm × 2 mm × 0.625 mm wafer with a gravity of 44.79 μN, as shown in Figure 11b. The volume of the capillary bridge was 0.9 μL. The connection between the probes and the wafer was established by the capillary bridge due to the movement of the three-finger microgripper. Then, the probes moved upward at a speed of 20 μm/s through the three-finger microgripper. The wafer with a size of 2 mm × 2 mm × 0.625 mm was picked up successfully. However, due to the large size of the wafers, a change in the wafer position was observed during the pickup of the wafer. Distilled water with a volume of 1.2 μL was also placed at the target position. Finally, the wafer with a size 2 mm × 2 mm × 0.625 mm was released in the target position by the three-finger microgripper upward motion successfully.

## 5. Conclusions

In the present study, the capillary force and the capillary rise height in three probes and a plate were investigated. The capillary model was established in the Surface Evolver environment using the energy minimization method. The capillary forces gained from the capillary bridge were measured using the experimental platform. The maximum capillary forces obtained from the capillary bridge model and the experiment were 95.5 μN and 96.0 μN, respectively. The agreement between the capillary bridge model results and the experiment results was excellent. The influences of the separation distance, capillary bridge volume, radial distance between probes, plate contact angle and probe side contact angle on the capillary force and the capillary rise were investigated based on the capillary model. The effect of various factors such as separation distance, capillary bridge volume and radial distance between the probes on the capillary force was not monotonic, caused by the edge effect. The effects of the plate contact angle and the probe side were monotonic. For *θ*_3_ = 100°, the capillary force reduced to −47.2 μN from the initial 166.9 μN when the plate contact angle increased to 150° from the initial 60°, and the capillary force reduced from 290.5 μN to 253.6 μN when the contact angle on the probe side was changed from 50° to 74° for *θ*_2_ = 45°. The results provide an effective strategy for controlling capillary forces generated by end-face-aligned three-finger microgrippers with probe side contact angles ≤ 90°. In addition, a wafer with sizes of 1 mm × 1 mm × 0.8 mm and 2 mm × 2 mm × 0.625 mm were picked up successfully using the three-finger microgripper.

## Figures and Tables

**Table 1:** The simulation parameters for capillary bridge models with different separation distances.

Parameters	Value
Radius of probes	0.25 mm
Radial distance between probes	0.2 mm
Side contact angle of the probe	80°
Contact angle of the plate	100°
Surface tension	67.4 mN/m

**Table 2:** The simulation parameters for capillary bridge models with different capillary bridge volumes.

Parameters	Value
Radius of probes	0.25 mm
Side contact angle of the probe	80°
Separation distance	0.3 mm
Contact angle of the plate	100°
Surface tension	67.4 mN/m

**Table 3:** The simulation parameters for capillary bridge models with different radial distances.

Parameters	Value
Radius of probes	0.25 mm
Side contact angle of the probe	80°
Contact angle of the plate	100°
Separation distance	0.3 mm
Surface tension	67.4 mN/m

**Table 4:** The simulation parameters for capillary bridge models with different plate contact angles.

Parameters	Value
Radius of probes	0.25 mm
Volume of capillary bridge	0.3 μL
Separation distance	0.3 mm
Radial distance between probes	0.2 mm
Surface tension	67.4 mN/m

**Table 5:** The simulation parameters for capillary bridge models with different probe side contact angles.

Parameters	Value
Radius of probes	0.25 mm
Volume of capillary bridge	0.3 μL
Separation distance	0.3 mm
Radial distance between probes	0.2 mm
Surface tension	67.4 mN/m

## Data Availability

The data presented in this study are available on request from the corresponding author.
